# Must We Vaccinate the Most Vulnerable? Efficiency, Priority, and Equality in the Distribution of Vaccines

**DOI:** 10.1111/japp.12588

**Published:** 2022-05-26

**Authors:** Emma J. Curran, Stephen D. John

**Affiliations:** ^1^ Faculty of Philosophy University of Cambridge Cambridge UK; ^2^ Department of History and Philosophy of Science University of Cambridge Cambridge UK

## Abstract

In this article, we aim to map out the complexities which characterise debates about the ethics of vaccine distribution, particularly those surrounding the distribution of the COVID‐19 vaccine. In doing so, we distinguish three general principles which might be used to distribute goods and two ambiguities in how one might wish to spell them out. We then argue that we can understand actual debates around the COVID‐19 vaccine – including those over prioritising vaccinating the most vulnerable – as reflecting disagreements over these principles. Finally, we shift our attention away from traditional discussions of distributive justice, highlighting the importance of concerns about risk imposition, special duties, and social roles in explaining debates over the COVID‐19 vaccine. We conclude that the normative complexity this article highlights deepens the need for decision‐making bodies to be sensitive to public input.

## 1 Introduction

The United Kingdom's Joint Committee on Vaccination and Immunisation (JCVI) is clear that ‘the first priorities for the COVID‐19 vaccination programme should be the prevention of mortality and the maintenance of the health and social care systems’.^1^ As such, it prioritises vaccinating those most vulnerable to COVID‐19, primarily through age‐based allocation. As with any decision about allocating scarce resources, this policy raises questions. One set of worries, explored by Giubilini, Savulescu, and Wilkinson, concerns the policy's ends: why prioritise the prevention of mortality, rather than saving life years?^2^ A second set concerns the means: how do we decide who counts as ‘vulnerable’, and how does this intersect with the JCVI's own concerns about equity?^3^ For example, recent work by Wrigley‐Field *et al*. suggests that targeting vaccinations on disadvantaged communities would do more good and be more equitable than an age‐based strategy.^4^ Finally, a third set of concerns centre around the relationship between these means and ends; perhaps the means of focusing on vulnerability does not best achieve the end of reducing mortality, as proposed by Rhodes who instead suggests a ‘transmission’ strategy, which prioritises vaccinating those who pose most risk, rather than the most at risk.^5^

Clearly, assessing arguments for practicable vaccine‐allocation systems is difficult, in part, because means and ends are deeply entangled. And, to make matters worse, because vaccines generate social as well as individual benefits, we cannot simply assume that principles for allocating scarce resources such as ventilators can be carried over to the distribution of vaccines. Further complexities arise because the ethics and epistemology of vaccination are intertwined; there is huge uncertainty about the effects of different policies, raising the question of how certain is ‘certain enough’ for action. Finally, any first‐order proposal must be consistent with laws and regulatory norms, making it unclear to whom proposals are directed. Perhaps vaccination policy should be guided by a concern for a broad range of capabilities, but ‘social’, as opposed to narrowly ‘medical’, outcomes are not necessarily within the remit of bodies such as the JCVI.^6^

This article takes a step back from current policies and first‐order proposals; instead, drawing on discussions in moral and political philosophy on the distribution of scarce resources, we construct a philosophical guide to the ethics of COVID‐19 vaccination, which clarifies both some of the acknowledged ethical issues around vaccination and how some apparently technical disputes implicitly reflect tensions between ethical principles. We do not make first‐order proposals about how vaccines ought to be distributed. We do, however, reach a second‐order conclusion: there should be much greater debate and transparency about vaccine‐allocation decisions.

This may all seem rather familiar. However, the value of our work is in its implications. You might think that going ‘deeper’ would resolve some of the problems implicit in unstructured lists of ‘ethical considerations’; we might be able to provide a principled account of what to do when principles like ‘maximise benefit’ and ‘equal concern’ conflict.^7^ We suggest the opposite. Indeed, the deeper we go, the more ambiguities and complexities we encounter, the more principles we uncover, and the less plausible it is to distinguish ‘scientific’ or ‘technical’ concerns from ‘ethical’ ones. We do not pretend it would be easy to open up every vaccine‐allocation decision to the degree of ethical scrutiny we think possible. Still, knowing what the ethical issues are both deepens the demand that decision‐making bodies are sensitive to public input, and, crucially, provides frameworks for structuring such deliberation.

Three clarifications are in order. First, our focus on the distribution of COVID‐19 vaccines may seem odd, because COVID‐19 vaccination programmes are well under way. However, thinking about these policies allows us to better respond to current and upcoming problems with COVID‐19 vaccination, for example over ‘booster’ programmes or vaccinating children. It also allows us to rethink other controversial cases of vaccination, such as the MMR or HPV vaccines.^8^ Second, much of the literature around vaccination revolves around whether, in virtue of the risks the unvaccinated pose to others, we can force people to be vaccinated.^9^ Whilst we agree that concerns about risk are important, our concern is less with the vaccine hesitant and more with the competing claims of the vaccine willing. Third, the global distribution of vaccines is ethically pressing and pragmatically relevant.^10^ To contain our discussion, we will, however, focus on the national scale, with a specific focus on the United Kingdom. We are, we admit, uncertain about the implications of our work for the global context, insofar as the core problem we explore – deep value‐disagreement over vaccine distribution – is likely even starker in global contexts.

In Section 2, we distinguish three general principles to distribute goods and two ambiguities in spelling them out. Combining these, we generate five plausible principles for vaccine distribution. Section 3 argues that we can understand actual debates around the COVID‐19 vaccine as reflecting disagreements over these *prima facie* plausible principles. In Section 4, we introduce a second source of complexity, by moving attention away from distributive justice to concerns about risk and social roles. In Section 5, we argue that there is no simple way of specifying, combining, or ranking these principles, discussing the implications in our conclusion.

## 2 The Central Planner

Imagine the following stylised version of policymakers' problem in the COVID‐19 pandemic:

A policymaker has a supply of vaccines. She knows that these vaccines are highly effective at stopping patients from developing symptoms, probably effective at stopping transmission of disease, and with minor or rare side‐effects. Unfortunately, the supply of vaccines is lower than the number of people who would plausibly benefit from being vaccinated.

Of course, various proposals were made in response to this challenge. For example, a report for the German Federal Ministry of Health suggested three goals (in order of priority): prevention of COVID‐19 hospitalisations and deaths; protection of persons with an especially high work‐related risk; and prevention of transmission in environments with a high proportion of vulnerable individuals.^11^ Meanwhile, the United Kingdom's JCVI took a different approach, allocating vaccines largely on the basis of age and pre‐existing medical conditions.^12^ Plausibly, deciding between such policies requires us to ask a more fundamental question: which principles *ought* to guide the distribution of scarce resources? The philosophical literature on related problems suggests three main candidates^13^:

*Efficiency*: Distribute goods in such a way as to have the greatest aggregate benefit.

*Equality*: Distribute goods in such a way as to ensure maximal equality between persons.

*Priority*: Distribute goods in such a way as to ensure that the least well‐off are as well‐off as possible.

To illustrate, imagine we must choose between curing a small number of people of debilitating pain or curing a far larger number of people of non‐infectious sore throats. Efficiency might favour the latter course of action, as long as the aggregate benefits of curing many sore throats is larger than the aggregate benefits of curing a small number of people's debilitating pain. By contrast, Priority might imply that we ought to choose the former option, as it would most help those who are least healthy. And, in this case, Equality concerns plausibly also point towards treating debilitating pain; a state of affairs in which many people have sore throats and a small number of people are in full health seems more equal than one where a small number of people are in debilitating pain and a large number in full health. Priority and Equality concerns, however, can come apart; imagine we could cure sore throats, but there was no option to help those with debilitating pain. Equality suggests some reason to abstain from curing sore throats, as doing so would increase health inequality. Priority does not make this, arguably counter‐intuitive, recommendation.^14^

One next step would be to specify, compare, and maybe combine these principles to identify the ‘correct’ approach. It is now, for example, common to adopt a ‘weak’ prioritarianism, according to which, roughly speaking, we ought to distribute goods such as to bring about the greatest aggregate sum of priority‐weighted good. Advocates argue that this approach captures the most appealing aspects of Efficiency and Priority.^15^ Note that, rather than assume more sophisticated forms of Priority, we assume a lexical reading of the principle, as giving absolute (rather than weighted) priority to the least well‐off.

Our focused discussion of lexical Priority is not because we believe it is the most plausible interpretation of the principle, all things considered. Indeed, it likely is not. But our purpose in this article is not to specify the most plausible formulation of these three principles or to identify the one ‘correct’ or ‘true’ principle to govern distribution. Rather, we aim to highlight that commitments to these general, popular, and oft‐cited principles can be unpacked and realised in a variety of ways, indeed ways which may give rise to contradictory distributional advice. As such, we use simplistic principles, such as lexical Priority, as a means of capturing the general spirit of these distributional outlooks. In particular, we wish to explore two ambiguities in spelling out the three principles: first, with respect to which wellbeing‐related states they concern; and second, whether they are interpreted *ex ante* or *ex post*.

When we use each of these principles to assess a decision, we also make, often implicit, assumptions about which sorts of wellbeing‐related states we ought to promote.^16^ A commitment, for example, to ensuring maximal equality between people raises the question: equality in what? Likewise, in focusing on the worst‐off we must make an assumption about which dimensions of being badly off are most important. Finally, when we focus on bringing about the largest sum of aggregate benefits, our judgements are shaped by assumptions about which benefits are relevant.^17^

Perhaps the most obvious way of specifying these principles in the vaccination context is to focus on health‐related aspects or determinants of wellbeing. But, of course, there are different ways of conceptualising and measuring health‐related wellbeing states. As Giubilini, Savulescu, and Wilkinson note, we must choose between lives saved, life years, or Quality Adjusted Life Years (QALYs).^18^ The choice of health metric will have significant impact on the verdict the various principles give in decision instances.

To add further complexity, one might believe we ought to focus on overall wellbeing, understood to include not only individuals' health‐related wellbeing but also their economic wellbeing. Likewise, this choice is likely to have multiple implications; it might change which vaccination policy is expected to bring about the greatest sum of good or wellbeing in aggregate. For example, a social‐utility approach might lead us to favour vaccinating tourism‐sector employees, as the Greek government did.^19^ Similarly, such a social‐utility approach might redefine who counts as antecedently worst off, and, hence, as having the strongest demand on resources, according to Priority. And, of course, things might change again if, for example, we included not only health and economic wellbeing, but, say, a broader set of basic capabilities.^20^

Provided death is the worst possible health outcome, concern for Priority might be naturally reconciled with a focus on ‘lives saved’. On the other hand, given that Efficiency is easily grounded in a desire to bring about the most aggregate good, similar considerations may point to a choice of a metric like the QALY. But, of course, principles and metrics do not map onto each other straightforwardly; one might think that the death of a young person is worse for them than the death of an elderly person. If so, then the concerns grounding Priority might also point towards a metric which captures the number of years of life lost, such as QALYs. In fact, QALYs might prove to be insufficient; if we measure health outcomes in QALYs, then we will be indifferent between seven people losing five years of life and one person losing 35 years of life. However, Priority, plausibly, does not merely involve a concern for the number of life years lost, but the distribution of these years; it is simply worse for one person to lose 35 years of life than for seven people to lose five years. As such, Priority might point towards a metric sensitive to this concern, for example, by some form of age weighting of life years. So, it may be a mistake to assume – as, say, Emanuel *et al*. do – that a decision as to whether to aim at saving lives or saving life years is simply a decision about how to specify ‘maximizing benefit’; rather, the choice may reflect a very different conception of permissible aggregation.^21^

Conversely, metric choice will also impact each principle's plausibility. Consider a standard objection against utilitarianism: that, because it aggregates across separate persons, it might lead us to policies which provide a very small benefit to very many people over policies which provide a much larger benefit to fewer people. One way of understanding this objection is as proposing problems with Efficiency. But we might also avoid this charge by placing a strong limit on which aspects of wellbeing are ‘counted’; in doing a cost–benefit analysis of lockdown measures, for example, we might simply ignore the very small displeasure experienced by the millions of people who are denied their daily trip to Costa Coffee.^22^

To simplify discussion, we will simply assume our focus should be on health‐related aspects of wellbeing (until Section 5). Yet, even placing to one side ‘metric’ problems, we need to resolve a second, cross‐cutting distinction between *ex ante* and *ex post* perspectives. The distinction centres around the perspective one takes when assessing risky decisions.^23^ An *ex ante* assessment of an intervention focuses on its effects on individuals' chances of being harmed and benefitted (their ‘prospects’) whereas an *ex post* assessment focuses on the intervention's expected effects on the overall pattern of outcomes.

Two policies might have the same expected outcomes – so, be identical from an *ex post* perspective – but impose different risks of harm and chances of benefit on affected individuals – so, differ *ex ante*. For example, imagine a choice between an intervention which would reduce the very high risk of breast cancer associated with BRCA1/BRCA2 or an intervention which would reduce the moderate breast‐cancer risk associated with being over 50 years old. Plausibly, given that there are very many more women over 50 than with the BRCA1/2 genes, these interventions might be identical in terms of their expected effects on mortality and morbidity. Nonetheless, the two strategies clearly differ *ex ante*: one reduces a very high risk for a small number of people, whereas the other reduces the far lower risk of a much larger number.^24^

Likewise, two policies may look identical *ex ante*, but different *ex post*. For example, consider a choice between triennial screening, which reduces breast‐cancer risk a bit and carries a low chance of overdiagnosis, and biennial screening, which reduces breast‐cancer risk more and carries a higher chance of overdiagnosis. Imagine each woman is indifferent between these programmes, such that both programmes have similar effects on each individual's *ex ante* prospects. Despite this, the programmes might look very different *ex post*, because the first only benefits (a few) individuals, whereas the second both benefits (more) individuals and harms some others.^25^

The *ex ante* and *ex post* perspectives pull our distributive principles in very different directions. To see how the perspectives might differ with regard to Equality, consider recent concerns that there has been differential vaccine uptake between different social and ethnic groups.^26^ In this case, you might think that even though members of different groups may have ‘equal chances’ of receiving a vaccine, through equal access, this does not guarantee equal vaccine uptake, and, resultantly, equal health outcomes. So, it might seem that there is a distinction between ensuring *ex ante* equality in vaccine distribution and *ex post* equality.

However, there is further complication. In this case, it is very plausible to think that *ex post* inequality in vaccine distribution outcomes is excellent evidence that the *ex ante* likelihood of individuals getting the vaccine, across different social and ethnic groups, was unequally distributed. Perhaps, even though each individual had the same formal opportunity, and therefore in some sense ‘equal chances’ of getting the vaccine, they differed in their substantive opportunities. That is, despite having formal access to vaccines, historic and structural factors made it such that certain groups were less likely to uptake the vaccine. As such, the same vaccine intervention will have unequally affected the *ex ante* prospects of members of different ethnic and social groups.^27^ As such, you might think that, on both *ex ante* and *ex post* Equality grounds, we should put extra effort into identifying and encouraging members of ethnic minorities to come forward for vaccination.^28^

However, whilst this policy might reduce *ex post* inequality (and, on one account, *ex ante* inequality), the efforts involved might involve diverting resources away from other aspects of the vaccination drive. If so, respecting Equality might come into conflict with Efficiency. Consider concerns in the United Kingdom that in areas where ethnic minority uptake of vaccines was low, ‘spare’ doses were offered to younger, White people.^29^ This policy seems easily justified by Efficiency, but it was controversial, insofar as some felt it would have been better, on grounds of Equality, to divert resources to older ethnic minority groups.

In discussing such tensions, Schmidt *et al*. highlight the importance of prioritising historically and structurally marginalised groups given the unequal outcomes faced by population groups.^30^ Again, however, it is important here to be careful about how we understand the force of such concerns. On the face of it, this proposal is that we should value Equality over Efficiency. On closer inspection, however, the concerns are framed in terms of ‘the pandemic's vastly disparate impact, especially on worse‐off minorities', suggesting it is unclear whether their concern is with equality *per se*, or a more prioritarian focus.^31^

The *ex ante* and *ex post* distinction is also relevant to thinking through Priority. Consider a highly simplified example:

a policymaker must distribute vaccines amongst two groups. In the first group, each individual is at very high risk of a moderate harm, such as prolonged illness – indeed each is almost certain to incur illness. In the second, each individual faces an extremely tiny chance of a significant harm, such as death. Due to the size of the second group, we have good statistical reason to believe that exactly one person would die if left unvaccinated.

In this case, both options capture something important about giving priority to the worst off. By vaccinating the first group, we are paying special attention to those who have the worst prospects – those who almost certainly are going to have severe long‐term illness. On the other hand, if we vaccinate the second group, we are avoiding an outcome in which someone would be made the most badly off – she would die.

Decisions surrounding COVID‐19, of course, often do not look like this; the group who are most vulnerable to COVID also produce the worst *ex post* outcomes (assuming the greatest sum of deaths is the worst health outcome).^32^ However, whilst this case is not directly relevant to current discussions, we might face a structurally similar problem around future choices, given that children (currently typically unvaccinated, and, hence, at higher risk of contracting the virus) are more likely to develop ‘long COVID’ whereas older people (typically vaccinated, and, hence, at lower risk of contracting the virus) are more likely to die of COVID.

We can now distinguish five, *prima facie*, plausible principles for vaccine distribution:

- *Efficiency*: Distribute vaccines in such a way as to have the greatest aggregate benefit (measured, for example, in terms of COVID‐related morbidity or mortality).
- *Ex Post Equality*: Distribute vaccines in such a way as to ensure equality in terms of some relevant outcome (such as vaccination uptake or COVID mortality).
- *Ex Ante Equality*: Distribute vaccines in such a way as to ensure each has an equal chance of some vaccine‐related benefit (such as an equal opportunity to get vaccinated).
- *Ex Post Priority*: Distribute vaccines in such a way as to ensure that the least well‐off are as well‐off as possible (e.g. that deaths are minimised).
- *Ex Ante Priority*: Distribute vaccines in such a way as to favour helping those who are most at risk (e.g. those at highest risk of contracting COVID or those at highest risk of COVID‐related mortality).

Note we only list five principles here, rather than including an *ex ante* and *ex post* version of Efficiency. This is a tricky topic, but roughly, there is no important difference between *ex ante* and *ex post* Efficiency, at least in our case.^33^

As we have suggested, each of the five principles outlined has some *prima facie* plausibility, and we suggest that this *prima facie* plausibility explains why so many first‐order arguments about the ethics of vaccination stress a plurality of ethical considerations. It might also explain why in practical debates over vaccination distribution, there seems to be genuine disagreement over what policy would best respect the broad slogans of ‘Equality’ and ‘Priority’. What we hope to have noted, however, is that the relationship between fundamental principles and specific recommendations is even more complex than it might seem; not only are there challenges in specifying principles, but these debates intersect with concerns about the relationship between Priority and Equality and issues about the moral relevance of risk.

You might, however, think that whilst such concerns are theoretically interesting they are practically empty, because the choice between principles does not make much practical difference. We will now argue that this is a mistake.

## 3 Principles in Practice

The United Kingdom's JCVI has primarily focused on an age‐based system for resource allocation, on the basis of the strong correlation between age and COVID‐associated mortality.^34^ On the face of it, this strategy seems to align with at least three of the principles above. First, helping the most at risk is recommended by *ex ante* Priority. Second, *ex post* Priority seems to imply we should reduce overall mortality, under the assumption that death is the worst outcome, and vaccinating the most vulnerable may seem the best strategy for this end. Third, you might think that reducing death rates is clearly the best way of improving overall population outcomes, as recommended by Efficiency. So, apparently, despite our philosophical carping, the only real problem is how to build equality concerns into the JCVI's approach. In turn, the JCVI considered this concern, stressing that its approach did, if only indirectly, help disadvantaged groups and stressing the importance of considering equity issues in vaccine delivery.^35^

However, this appealing chain of reasoning is doubly flawed. First, even if vaccinating the most at risk does most to reduce mortality, it does not follow that it is thereby the best strategy for improving overall population health. Although the young are less likely to die of COVID than the elderly, they can still suffer badly, for example, through contracting ‘long COVID’. Conversely, simply because they are old, those most at risk of COVID may ‘gain’ few life years from being vaccinated. We might adopt different policies if we care about minimising deaths, as Priority apparently suggests, and if we care about maximising QALYs, as Efficiency might suggest.^36^ Epidemiological data suggest that, at least in an initial programme, a policy of favouring the elderly over the young would both maximise lives saved and QALYs saved.^37^ However, it is worth noting that this congruence is not guaranteed – indeed, Rhodes questions if it were true even of the initial programme.^38^

Second, consider, again, the JCVI's stated end – to reduce mortality and ‘protect’ the NHS – and its strategy of vaccinating the most vulnerable. Assume that the epidemiological data are correct and that reducing overall mortality can be justified on both Efficiency and *ex post* Priority grounds. The chosen means to this end, vaccinating those at greatest risk, seems justifiable in the context of *ex ante* Priority. Unfortunately, it is not clear that the chosen means will, in fact, achieve the stated ends. It is possible that preferentially vaccinating younger, less vulnerable people will lead to lower overall morbidity and mortality than vaccinating older, more vulnerable people, given that younger people may be more likely to transmit the disease. Indeed, something like this is true in the case of some flu vaccines.^39^ Furthermore, when the JCVI considered the introduction of a ‘booster’ programme in mid‐2021, there was at least some (model‐derived) evidence that a strategy of focusing boosters on younger age groups would be preferable – for transmission reasons – to vaccinating those at greater ‘direct’ risk.^40^

If so, *ex ante* and *ex post* Priority will come apart. Of course, vaccinating the young brings some benefit to each older person, by reducing her risk of contracting disease. However, for any specific older person, she would be even better off were she to receive the vaccination directly. As such, *ex ante* Priority, which tells us to do the thing which will most improve the health prospects of those with the worst health prospects, will still tell us to vaccinate the elderly and vulnerable. *Ex post* Priority, however, will tell us to prevent the worst outcome in which the most people die of COVID, by vaccinating younger, less at risk people to block transmission.^41^

There is, then, ambiguity over how best to interpret the UK policy; what were the underlying justifications? In a meeting which first considered possible vaccination strategies and which considerably influenced later policy, a decision was made to focus on strategies which reduced mortality, and it was decided that a ‘risk‐based’ approach to distribution best met that end.^42^ Note, however, that there were discussions of adopting different goals – focusing on QALYs gained^43^ – and different strategies – focusing on ‘transmission groups’.^44^ In the terms above, the JCVI chosen ends of minimising mortality can be viewed as adopting an *ex post* Priority focus, rather than Efficiency concern. Although their chosen means can be reconciled with *ex ante* Priority, it was chosen as a strategy for minimising mortality, rather than as reflecting independent ethical concern.

The JCVI's reasoning behind their chosen ends and means was explicitly linked to available data: it was relatively easier to identify policies which would minimise mortality than policies which would maximise QALYs saved; and relatively easier to assess the effects of age‐based, over transmission‐based, vaccination strategies. Therefore, the group was keen to stress that this approach was ‘based on scientific principles’ and not ‘ethical considerations’ which they suggested should be handled at some other stage.^45^ Please note that we are not proposing that the JCVI made the wrong ethical decision; nor that members were unaware that their decisions had ethical consequences. Indeed, issues around equity and equality were often discussed at later meetings, and their most important report on vaccine allocation listed three core ethical goals for allocation: to benefit and reduce harm, to be fair and transparent, and to address health inequalities.^46^

However, it remains true that, to a very large extent, the JCVI's decisions were presented as simply ‘following’ from aspects of scientific understanding, without clarifying the relationship to its vague ethical principles. Clearly, at a theoretical level, this is problematic. Even if we can have greater scientific certainty of the effects of A on B than of the effects of C on D, no ‘scientific’ principle tells us that we must choose the first strategy. Rather, this is an ethical judgement, and a contestable one at that; we might be more certain that giving our money to Charity 1 will improve the orchestra than we are that giving our money to Charity 2 will reduce world hunger, but we have greater ethical reason to give our money to Charity 2. Similarly, we might be more certain how to reduce mortality than how to maximise QALYs, but it does not follow we must then adopt the former aim.

The failure to clarify the relationship between the JCVI's purported ethical concerns and the scientific data, by making it unclear which specific ethical principles underlay its policy, gave rise to (at least) two practical problems. One problem concerns the relevance of new evidence. As we noted above, to a large extent, the decision to adopt a ‘risk‐based’ *versus* ‘transmission’ strategy was based on a lack of evidence about the effects of the vaccine on transmission. Ignorance allowed the JCVI to sidestep the difficult question of whether there might be *ex post* reasons to vaccinate those who are not most vulnerable *ex ante*. However, as we noted above, later, when considering possible ‘booster’ programmes, there was more evidence on transmission.^47^ By this point, however, members of the committee felt that it would be ‘confusing’ to the public to change strategy. In effect, by not properly articulating underlying ethical issues, the JCVI closed off its own scope for flexibility.

Second, there is a problem of ‘unwarranted public support’. The choice of an *ex ante* Priority strategy was simply a tool for ensuring a particular *ex post* end, of reducing mortality. However, we suggest that part of the public understanding and appeal of this policy might have been grounded in an independent moral concern, that there is something inherently valuable in responding to the most vulnerable. In turn, then, this potential gap can lead to a problematic situation where what, for some audiences, would be a troubling change in ethical aims was presented as if simply a technical decision. For example, in January 2021, the JCVI announced an apparently technical tweak to its strategy; whilst still distributing vaccines primarily on the basis of age, instead of leaving three weeks between first and second doses, the NHS would leave 12 weeks.^48^ The reasoning was that the longer the gap between doses, the greater the number of people who receive at least a first dose, and hence have some protection against the disease, which would, in turn, have a greater overall effect on mortality and morbidity. This may seem sensible, but it is tantamount to not helping the most vulnerable as much as we could have done. In effect, it slightly reduces the effectiveness of the vaccination programme for each of the older people who received a first dose for the sake of reducing overall mortality. So, assessing this strategy requires us to determine whether we should prioritise the vulnerable beyond a concern for maximising outcomes.

Note a potential complication: you might think that, once the elderly have received a first dose, they are no longer the ‘most vulnerable’ in society, so there is an *ex ante* Priority argument for spreading the gap more widely. Roughly, as soon as we provide a first dose for the oldest unvaccinated age cohort, our *ex ante* Priority concern should automatically shift to the second oldest unvaccinated age cohort. We do not aim to resolve this question. However, the need for detailed analysis of such topics is more grist to our mill, as it is more evidence that in not articulating the ethical bases of its decisions more clearly, the JCVI left open space for confusion.

Having introduced multiple levels of complexity into thinking through the distribution of vaccines, and having suggested that the actual pandemic response showed real tensions between these principles, it may seem that the natural next thing to do is to further refine, specify, and compare the principles, aiming to propose one to guide policymaking. In the next section, however, we continue to highlight sources of complexity.

## 4 Beyond the Central Planner

So far, we have assumed a standard framework from ethics and political philosophy, where we think about distributive questions as if from the perspective of a central planner, who aims at ensuring distributive outcomes. Arguably, this is also the perspective which dominates epidemiology and health economic analysis, where we focus on aggregation and distribution across populations. In this section, we now explore a set of worries, which fit less well within this ‘God's eye’ framework, grounded on the ethically ‘thick’ obligations which individuals and policymakers possess. Specifically, we focus on two topics: the imposition of harm and reasonable risk, reciprocity, and social roles.

First, consider concerns about side‐effects of vaccination. Some of the vaccines against COVID‐19 have exceedingly rare side‐effects. Several states responded by placing significant limitations on these vaccines' use, as in the JCVI's decision to limit use of the AstraZeneca vaccine in younger populations.^49^ These decisions do not seem to make much sense in terms of any of the principles outlined above; given the rarity of the side‐effects, their existence makes only an infinitesimal difference to any individual's *ex ante* prospects and hardly changes the overall *ex post* balance of costs and benefits.

Here is one possible account of what underpins such concerns. In general, we often distinguish between doing harm and failing to prevent harm, viewing the former as morally worse than the latter. This asymmetry is baked into medical ethics, where a core principle of good clinical practice is ‘non‐maleficence’, or ‘first, do no harm’.^50^ Let us assume that there is a strong, but defeasible, injunction against medical interventions which cause harm to some, even if they would be outweighed by ‘benefits’ to others. Assume, further, that these principles also apply to social decision‐making. If so, potentially, we get a sixth principle to guide vaccine distribution problems:

vi *Do No Harm*: Do not distribute vaccines if there is a sufficient chance that they will cause serious harm, even if the expected aggregate benefits far outweigh the possible harms.

Second, consider the question of whether healthcare workers ought to be prioritised for vaccination.^51^ Plausibly, this policy gains support from multiple perspectives, given the high risks they suffer and the high chance they will transmit the virus. However, there may seem to be another, more complex, reason to favour vaccinating healthcare workers: they are performing a socially admirable role, and it is unreasonable to expect them to take on increased risk. That is to say, such responses seem linked to notions of ‘reciprocity’, which, interestingly, have been central to some accounts of vaccine ethics but are completely missing from others (most obviously, the JCVI principles listed above).^52^

Similarly, consider demands from teachers in the United Kingdom that they receive vaccines before returning to the classroom. Many experts claimed that there was no compelling public health reason to accede to these demands. However, we can, again, understand the teachers' demands less in terms of large‐scale distributive concerns, and more as a claim that it would be unreasonable to expect them to take on avoidable, excess risk as part of discharging their socially valuable, role‐related obligations. Such claims cannot be reduced to concerns solely about what would be ‘best’ – from the viewpoint of health outcomes or social utility more generally. Rather these concerns seem to be underpinned by social understandings of the meaning and importance of certain forms of work. These concerns are, admittedly, vague, and, indeed, it is interesting that different accounts of reciprocity identify very different groups as deserving of special attention. But these considerations apparently suggest yet another principle:

vii *Social Meaning*: Distribute vaccines in such a way as to reflect concerns about what level of risk it would be reasonable to expect people to run for the sake of performing their work.

Just as we noted possible tensions between principles i to v in the previous section, principles vi and vii could mandate courses of action which look odd from other perspectives; for example, it may well be that vaccinating firefighters would be more effective on all five principles discussed above than vaccinating delivery drivers, but, still, delivery drivers might be viewed as having some claim which firefighters do not. Furthermore, both principles are in tension with other plausible principles: for example, rigid application of Do No Harm might clash with individuals' ability to make their own medical choices.

## 5 Who Wins?

In this article, we have both complicated various principles explicitly discussed in debates over vaccine distribution and suggested other principles which might – sometimes implicitly – guide those debates. However, you might reasonably want more than a high‐level redescription of debate: you might want to know the correct account of how to distribute vaccines. This is a reasonable demand, but one which faces five significant challenges.

The first is that, as we already noted in Section 2, there are complicated issues around specifying the goods we care about distributing. Even within the domain of health‐related aspects of wellbeing, we must, for example, choose between a focus on lives or on life years. In turn, these issues create challenges for thinking about the relationship between mid‐level principles and broader philosophical theorising. For example, even if one is committed to a utilitarian principle that we should maximise aggregate wellbeing, it does not follow that one must endorse Efficiency, at least as long as that principle is couched solely in terms of health‐related goods, rather than overall wellbeing.

The second set of concerns revolve around operationalising Equality and *ex ante* Priority. When we compare outcomes in terms of inequality, we typically do so in terms of differences between groups – say, different ethnic minorities. Similarly, when we talk of individuals as being ‘vulnerable’, we do so in virtue of their group membership – say, the elderly or the immunocompromised. However, the ways we have of grouping people are contestable. For example, even if we think that we should focus on the ‘most at risk’, we can operationalise this concern using age‐based or geographical‐based distribution. So, someone who is in the ‘most at risk’ category in the first approach – say, a random 80‐year‐old – may not be ‘most at risk’ according to the second – say, if she lives in a leafy suburb. In turn, one way of thinking about this choice is solely in terms of the outcomes; maybe, as Wrigley‐Field *et al*. argue, we do more good and reduce *ex post* inequality best by using the second approach.^53^ However, plausibly, our choice of reference class is not just about outcomes but reflects concerns about how people ended up vulnerable, reflecting broader social justice concerns (as in Schmidt *et al*.).^54^ The specification of principles requires independent ethical theorising about which reference classes matter.^55^

The third complication is that, given both the novelty of the COVID‐19 virus and the speed at which vaccines have been developed, there is significant empirical uncertainty. We might simply lack the knowledge to know whether, say, vaccinating the young or vaccinating the elderly will have the greatest effect on population health outcomes. This, of course, was the situation faced by the JCVI. We need a further theory of how to think about the application and relevance of principles when there is uncertainty.

Fourth, the effects of vaccination on transmission exacerbate this epistemic uncertainty about predicting the effects of programmes (both *ex ante* and *ex post*) and create ethical complexity. Plausibly, if vaccination does prevent transmission, then we have not only prudential but ethical reasons to be vaccinated, because failures to vaccinate may be tantamount to imposing risks on non‐consenting others.^56^ This may be important from the *ex ante* perspective; if we were thinking about the distribution of scarce medical resources, such as ventilators, it would seem inappropriate to distribute those goods in a way which worsened individuals' prospects. But, if we think that people have an ethical obligation to vaccinate, it might be permissible to distribute vaccines to people for whom vaccination is not in their interests, because it is in others' interests that they vaccinate. Consider, for example, the febrile debate over whether we should encourage schoolchildren to vaccinate, even if this is not in their own interests, as a way of stopping community transmission.^57^ Any account of the ethics of vaccination needs to intersect with debates over permissible risk imposition and defensive harm.

Fifth, we have a problem of understanding the relationship between distributive concerns and agentive duties and social meanings. It is tempting to think of some of the issues we raised in Section 4 around side‐effects and social roles as ‘feasibility’ constraints – as if principles of distributive justice tell us how we really ought to distribute vaccines – but we might need to adopt different approaches given public sentiment. We think this approach understates the potential importance of these concerns. In articulating a concern that vaccines might impose harm, someone is articulating a distinctively ethical concern, rather than some mere preference. There is a genuine question as to whether we should think of state action as simply a giant exercise in central planning, as opposed to an attempt to enact and respect thick, socially shaped norms and duties.

## 6 Conclusion

This article has argued that there is likely to be substantial, reasonable disagreement over how best to allocate vaccines. Even if all reasonable people agree that it would be wrong to leave vaccine allocation solely to market mechanisms, they might disagree over how we ought to specify and balance concerns about Priority, Equality, and Efficiency. In this kind of case, it is less important that policy is guided by ‘the’ correct set of principles – if, indeed, there is any such thing – than that decisions are justifiable: that they are formed in ways which are informed by, and sensitive to, the wide range of ethically relevant considerations. In turn, this condition has important institutional implications: it is important that institutions which decide on vaccine distribution are structured to allow input from a wide variety of perspectives, that their deliberation is open and transparent, and that they offer justifications for the conclusions they reach. In this way, even if there is disagreement over whether they have identified the correct principles, we can be assured that their decisions are reasonable.

These may seem, in the words of one reviewer, rather ‘bog‐standard’ recommendations. After all, many commentators agree that, in virtue of reasonable disagreement, resource allocation may require public oversight, and, as we have acknowledged, there is already no shortage of lists of proposed principles for vaccine allocation. What, then, does our deep dive into the philosophical literature add to these debates? In conclusion, we make three remarks.

First, we hope to have clarified that there may be many more ethical principles and concerns at stake in vaccine‐allocation decisions than are often explicitly recognised. Part of this has involved highlighting the roles of Do No Harm and Social Meaning in explaining disagreements over vaccine distribution. Similarly, within the more standard distributive framework, we have stressed the various, importantly different ways of conceiving of fairly standard considerations such as Efficiency, Equality, and Priority. And in doing so, we have hoped to draw attention to the various ethical considerations which might inform and justify both the purported goals and means of vaccine policies; for example, by highlighting that a focus on those at risk may be more than a tool to maximise outcomes, but it may reflect independent moral concern such as those who underpin *ex ante* Priority. At the very least, we suggest that these proposals should make us rethink debates between ‘risk’ and ‘transmission’ strategies as reflecting more than just epistemic disagreement over the best means to our ends.

Second, and related, we have explored how various apparently technical decisions, not only over transmission strategies but how to operationalise risk strategies or the correct spacing between doses, can be seen as incorporating or reflecting substantive ethical commitments. A standard division of labour, where some people do ‘the science’ and others do ‘the ethics’, is insufficient to capture the ways in which technical decisions are always implicitly ethically weighted. This implies that any opening up of decisions must be deep, not merely treating ethical principles as an ‘input’ to decision‐making.

Third, and most generally, as well as clarifying terms of discussions and the need for a wide scope of discussion, we have also strengthened the motivation for discussion. A deep dive into the philosophical literature does not mitigate the acknowledged problems of presenting policymakers with unstructured lists of principles, but deepens it; there are many more plausible principles and concerns than we might recognise. Plausibly, there are many more we have not even considered. The need for public engagement is not merely some contingent fact, which might, in principle, be solved by doing more philosophy; rather, it is the proper conclusion to draw from doing philosophy.^58^

## NOTES

1 UK Joint Committee on Vaccination and Immunisation. 2020a. “Joint Committee on Vaccination and Immunisation: Advice on Priority Groups for COVID‐19 Vaccination.” p. 10. https://assets.publishing.service.gov.uk/government/uploads/system/uploads/attachment_data/file/950113/jcvi‐advice‐on‐priority‐groups‐for‐covid‐19‐vaccination‐30‐dec‐2020‐revised.pdf. Accessed 28 August 2021.

2 Giubilini, A., J. Savulescu, and D. Wilkinson. 2021. “Queue Questions: Ethics of COVID‐19 Vaccine Prioritization.” *Bioethics* 35(4): 348–55.

3 UK Joint Committee on Vaccination and Immunisation. 2021a. “Annex A: COVID‐19 Vaccine and Health Inequalities: Considerations for Prioritisation and Implementation.” https://www.gov.uk/government/publications/priority-groups-for-coronavirus-covid-19-vaccination-advice-from-the-jcvi-30-december-2020/annex-a-covid-19-vaccine-and-health-inequalities-considerations-for-prioritisation-and-implementation. Accessed 20 January 2022.

4 Wrigley‐Field, E., M. V. Kiang, A. R. Riley, M. Barbieri, Y. Chen, K. A. Duchowny, E. C. Matthay, *et al*. 2021. “Geographically Targeted COVID‐19 Vaccination is More Equitable and Averts More Deaths than Age‐Based Thresholds Alone.” *Science Advances* 40(7): eabj2099. See also Schmidt, H., P. Pathak, T. Sonmez, and M. U. Unver. 2020. “Covid‐19: How to Prioritize Worse‐Off Populations in Allocating Safe and Effective Vaccines.” *British Medical Journal* 371: m3795.

5 Rhodes, R. 2021. “Justice in COVID‐19 Vaccine Prioritisation: Rethinking the Approach.” *Journal of Medical Ethics* 47: 623–31.

6 See Millar, M. R., Y. Gourtsoyannis, and A. Jayakumar. 2022. “Ethics of Vaccination: Should Capability Measures be Used to Inform SARS‐CoV‐2 Vaccination Strategies?” *British Journal of Clinical Pharmacology* 88(1): 47–55.

7 National Academies of Science, Medicine and Engineering. 2020. “A Framework for Equitable Allocation of Vaccine for the Novel Coronavirus.” https://www.nationalacademies.org/our-work/a-framework-for-equitable-allocation-of-vaccine-for-the-novel-coronavirus. Accessed 28 August 2021.

8 Dawson, A. 2011. “Vaccination Ethics.” *Public Health Ethics* 201(1): 143–53.

9 See, for example, Flanigan, J. 2014. “A Defense of Compulsory Vaccination.” *HealthCare Ethics Committee Forum* 26(1): 5–25.

10 Emanuel, E. J., G. Persad, A. Kern, A. Buchanan, C. Fabre, D. Halliday, J. Heath, *et al*. 2020. “An Ethical Framework for Global Vaccine Allocation.” *Science* 369(6509): 1309–12.

11 Joint position paper of the Standing Committee on Vaccination (STIKO), the German Ethics Council, and the National Academy of Sciences Leopoldina. 2020. “Recommendations for Fair and Regulated Access to a COVID‐19 Vaccine.” https://www.leopoldina.org/en/press-1/press-releases/press-release/press/2750/. Accessed 20 January 2022.

12 UK Joint Committee on Vaccination and Immunisation 2020a op. cit.

13 In distinguishing these principles, we draw on familiar work in moral and political philosophy: for discussion, see, *inter alia*: Norcross, A. 1997. “Comparing Harms: Headaches and Human Lives.” *Philosophy and Public Affairs* 26(2): 135–67; Parfit, D. 2002. “Equality versus Priority.” In *The Ideal of Equality*, edited by M. Clayton and A. Williams, 81–125. New York: Palgrave Macmillan; Wolff, J. 2007. “Equality: The Recent History of an Idea.” *Journal of Moral Philosophy* 4(1): 125–36; Hirose, I. 2014. *Moral Aggregation*. Oxford: Oxford University Press; Otsuka, M., and A. Voorhoeve. 2018. “Equality versus Priority.” In *The Oxford Handbook of Distributive Justice*, edited by S. Olsaretti, 65–85. Oxford: Oxford University Press. For discussion around health and health care specifically, see: Daniels, N. 2007. *Just Health: Meeting Health Needs Fairly*. Cambridge: Cambridge University Press; Bognar, G., and I. Hirose. 2014. *The Ethics of Health Care Rationing*: *An Introduction*. Abingdon: Routledge; Hausman, D. M. 2015. *Valuing Health: Well‐being, Freedom, and Suffering*. New York: Oxford University Press.

14 Parfit op. cit.

15 Otsuka and Voorhoeve op. cit.

16 In this discussion, we draw on what used to be called the debate over the ‘metric’ of distributive justice; for useful overviews and contributions, see: Sen, A. 1980. “Equality of What?” In *Tanner Lectures on Human Values*, Vol. 1, edited by S. McMurrin, 197–220. Cambridge: Cambridge University Press; Cohen, G. A. 1989. “On the Currency of Egalitarian Justice.” *Ethics* 99(4): 906–44; Daniels, N. 1990. “Equality of What: Welfare, Resources, or Capabilities?” *Philosophy and Phenomenological Research* 50: 273–96.

17 John, S. D., and E. J. Curran. 2021. “Costa, Cancer and Coronavirus: Contractualism as a Guide to the Ethics of Lockdown.” *Journal of Medical Ethics*. https://doi.org/10.1136/medethics-2020-107103.

18 Giubilini *et al*. op. cit.

19 Smith, H. 2021. “Aegean Islands Aim to become First Fully Vaccinated Areas of Greece.” *Guardian*, April 6, 2021. https://www.theguardian.com/world/2021/apr/06/aegean-islands-to-become-first-covid-free-areas-of-greece. Accessed 28 August 2021.

20 Millar *et al*. op. cit.

21 Emanuel, E. J., G. Persad, R. Upshur, B. Thome, M. Parker, A. Glickman, C. Zhang, *et al*. 2020. “Fair Allocation of Scarce Medical Resources in the Time of Covid‐19.” *New England Journal of Medicine* 382(21): 2049–55.

22 John and Curran op. cit.

23 Again, there is a vast literature on this topic. For some suggestions which have shaped our discussion, see: Frick, J. 2015. “Contractualism and Social Risk.” *Philosophy and Public Affairs* 43(3): 175–223; John, S. D. 2014. “Risk, Contractualism, and Rose's ‘Prevention Paradox.'” *Social Theory and Practice* 40(1): 28–50; Steuwer, B. 2021. “Contractualism, Complaints, and Risk.” *Journal of Ethics and Social Philosophy* 19(2): 111–47; Fleurbaey, M., and A. Voorhoeve. 2013. “Decide as You Would with Full Information.” In *Inequalities in Health: Concepts, Measures, and Ethics*, edited by N. Eyal, S. A. Hurt, O. F. Norheim, and D. Wikler, 113–28. New York: Oxford University Press.

24 John op. cit.

25 John, S. D., and J. Wu. 2022. “‘First, Do No Harm’? Non‐Maleficence, Population Health and the Ethics of Risk.” *Social Theory and Practice*. https://doi.org/10.5840/soctheorpract2022218152.

26 Razai, M. S., T. Osama, D. G. McKechnie, and A. Majeed, 2021. “Covid‐19 Vaccine Hesitancy among Ethnic Minority Groups.” *British Medical Journal* 372: n513.

27 Note that, strictly, we could think of equality of opportunity here in two ways: in terms of an equal probability of getting vaccinated (regardless of agential choice) and in terms of an equal opportunity to be benefitted (subject to agential choice). Exploring this option further would simply add to our argument that we need to be very careful how we specify principles. Thanks to an anonymous reviewer for pointing this out.

28 UK Joint Committee on Vaccination and Immunisation 2021a op. cit.

29 Nevett, J. 2021. “Covid‐19 Vaccines: Is It OK to Get a Leftover Jab?” BBC News. https://www.bbc.co.uk/news/world-55841017. Accessed 10 July 2021.

30 See Schmidt *et al*. op. cit.; Schmidt, H. 2020. “Vaccine Rationing and the Urgency of Social Justice in the Covid‐19 Response.” *Hastings Center Report* 50(3): 46–49.

31 Schmidt *et al*. op. cit., p. 3.

32 It should be noted, however, that something like the thought experiment might be going on in the COVID case if we think that the more premature a death, the worse a health outcome it is. In this case, whilst prioritising vaccinating the elderly may serve *ex ante* Priority, it may lead to an outcome in which some young people die who otherwise would not have if given the vaccine – thereby conflicting, starkly, with *ex post* Priority.

33 Thanks to Dan Hausman for useful discussion on this point. Roughly, the aggregate sum of prospects associated with an intervention is, in our simplified case, equal to the expected outcome of the intervention. As Efficiency is a straightforwardly aggregative principle, its evaluation of the (*ex ante)* prospects of an intervention will coincide with its evaluation of an intervention's (*ex post)* outcomes.

34 UK Joint Committee on Vaccination and Immunisation 2020a op. cit.

35 UK Joint Committee on Vaccination and Immunisation 2020a op. cit.; UK Joint Committee on Vaccination and Immunisation 2021a op. cit.

36 Note that there is a link here to debates around lockdown, where some commentators worried that a single‐eyed focus on saving lives led to lockdowns which ‘cost’ more in terms of their *overall* health‐related effects.

37 UK Joint Committee on Vaccination and Immunisation. 2020b. “Minute of the JCVI Extraordinary Meeting on COVID‐19 Immunisation.” https://app.box.com/s/iddfb4ppwkmtjusir2tc/file/729773393403. Accessed 1 September 2020.

38 Rhodes op. cit.

39 Galvani, A. P., T. C. Reluga, and G. B. Chapman. 2007. “Long‐Standing Influenza Vaccination Policy is in Accord with Individual Self‐Interest but Not with the Utilitarian Optimum.” *Proceedings of the National Academy of Sciences of the United States of America* 104(13): 5692–97.

40 UK Joint Committee on Vaccination and Immunisation. 2021b. “Minute of the COVID‐19 Sub‐committee Held Thursday 17 June 2021.” https://app.box.com/s/iddfb4ppwkmtjusir2tc/file/878925657397. Accessed 17 June 2021.

41 It is worth noting here that whilst the form of Priority we have been discussing is a lexical form, this does not mean it is fully non‐aggregative. As such, it is coherent to think *ex post* Priority would not be indifferent between a state of affairs with one death and a state of affairs with 100 deaths. Rather, *ex post* Priority can ask us not simply to prevent the worst possible outcome, death, but also the state of affairs which had the greatest number of deaths.

42 UK Joint Committee on Vaccination and Immunisation. 2020c. “Minute of the JCVI Extraordinary Meeting on COVID‐19 Immunisation Prioritisation.” https://app.box.com/s/iddfb4ppwkmtjusir2tc/file/680505467104. Accessed 7 May 2020.

43 Ibid., p. 15.

44 Ibid., p. 12.

45 Ibid., p. 16.

46 UK Joint Committee on Vaccination and Immunisation 2021a op. cit.

47 UK Joint Committee on Vaccination and Immunisation 2021b op. cit.

48 UK Joint Committee on Vaccination and Immunisation. 2021c. “Optimising the COVID‐19 Vaccination Programme for Maximum Short‐Term Impact.” https://www.gov.uk/government/publications/prioritising‐the‐first‐covid‐19‐vaccine‐dose‐jcvi‐statement/optimising‐the‐covid‐19‐vaccination‐programme‐for‐maximum‐short‐term‐impact. Accessed 13 January 2022.

49 UK Joint Committee on Vaccination and Immunisation. 2021d. “JCVI Statement on Use of the AstraZeneca COVID‐19 Vaccine.” https://www.gov.uk/government/publications/use-of-the-astrazeneca-covid-19-vaccine-jcvi-statement/jcvi-statement-on-use-of-the-astrazeneca-covid-19-vaccine-7-april-2021. Accessed 7 April 2021.

50 John and Wu op. cit.

51 UK Joint Committee on Vaccination and Immunisation 2020a op. cit.

52 For an example of such a response, see National Academies of Science, Medicine and Engineering. 2020. “A Framework for Equitable Allocation of Vaccine for the Novel Coronavirus.” https://www.nationalacademies.org/our-work/a-framework-for-equitable-allocation-of-vaccine-for-the-novel-coronavirus. Accessed 28 August 2021.

53 Wrigley‐Field *et al*. op. cit.

54 Schmidt op. cit.

55 John op. cit.

56 Flanigan op. cit.

57 UK Joint Committee on Vaccination and Immunisation 2021b op. cit.

58 The authors would like to thank the British Academy for funding this research. They would also like to thank the attendees of the work‐in‐progress seminar at the Rutgers Center for Population‐Level Bioethics for their helpful insights, alongside two anonymous reviewers for their thoughtful comments.

